# Involvement of *N*-methylpurine DNA glycosylase in resistance to temozolomide in patient-derived glioma cells

**DOI:** 10.1038/s41598-020-78868-0

**Published:** 2020-12-17

**Authors:** Gemma Serrano-Heras, Beatriz Castro-Robles, Carlos M. Romero-Sánchez, Blanca Carrión, Rosa Barbella-Aponte, Hernán Sandoval, Tomás Segura

**Affiliations:** 1 Research Unit, Complejo Hospitalario Universitario de Albacete, Laurel, s/n, 02008 Albacete, Spain; 2 Department of Neurology, Complejo Hospitalario Universitario de Albacete, Albacete, Spain; 3 Department of Anatomical Pathology, Complejo Hospitalario Universitario de Albacete, Albacete, Spain; 4 Department of Neurosurgery, Complejo Hospitalario Universitario de Albacete, Albacete, Spain; 5 Instituto de Investigación en Discapacidades Neurólogicas (IDINE), Facultad de Medicina, Universidad de Castilla-La Mancha, Albacete, Spain

**Keywords:** Translational research, Predictive markers, CNS cancer

## Abstract

Chemotherapy for high-grade astrocytic tumors is mainly based on the use of temozolomide (TMZ), whose efficacy is limited by resistance mechanisms. Despite many investigations pointing to *O*6-methylguanine-DNA-methyltransferase (MGMT) as being responsible for tumor chemo-resistance, its expression does not predict an accurate response in most gliomas, suggesting that MGMT is not the only determinant of response to treatment. In this sense, several reports indicate that *N*-methylpurine-DNA-glycosylase (MPG) may be involved in that resistance. With that in mind, we evaluated for the first time the degree of resistance to TMZ treatment in 18 patient-derived glioma cells and its association with MGMT and MPG mRNA levels. Viability cell assays showed that TMZ treatment hardly caused growth inhibition in the patient-derived cells, even in high concentrations, indicating that all primary cultures were chemo-resistant. mRNA expression analyses showed that the TMZ-resistant phenotype displayed by cells is associated with an elevated expression of MPG to a greater extent than it is with transcript levels of MGMT. Our findings suggest that not only is MGMT implicated in resistance to TMZ but MPG, the first enzyme in base excision repair processing, is also involved, supporting its potential role as a target in anti-resistance chemotherapy for astrocytoma and glioblastoma.

## Introduction

Gliomas are the most prevalent primary brain malignancies and encompass two principle subgroups: diffuse gliomas and gliomas showing a more circumscribed growth pattern (nondiffuse gliomas). These tumors arise from glial cells in the brain, particularly cells of oligodendroglial and astrocytic lineage. The principal symptoms of high grade gliomas are progressive neurological impairment, motor weakness, cognitive deficit, seizures and intracranial hypertension^1^. Historically, phenotypic histological characteristics classified gliomas into grade I (pylocitic astrocytoma), grade II (diffuse low grade glioma), grade III (anaplastic glioma) and grade IV (glioblastoma)^2^. According to the revised fourth edition of the WHO Classification of central nervous system tumors published in 2016, classification of diffuse gliomas has fundamentally changed: for the first time, these tumors, including diffuse astrocytoma, anaplastic astrocytoma, oligodendroglioma, oligoastrocytoma and glioblatoma, are now defined based on presence/absence of IDH mutation and 1p/19q codeletion^3^. The integration of both phenotypic and genotypic characteristics has important implications in prognosis and therapeutic approach. However, all malignant gliomas share the same properties such as uncontrolled cell proliferation, diffuse infiltration, apoptotic resistance, high angiogenesis and genomic instability^4^.

Treatment of patients for high grade astrocytomas is always interdisciplinary. After characterization by imaging techniques, maximal safe surgical resection of tumor mass is performed^5^, followed by radiotherapy and concomitant chemotherapy, mainly with temozolomide (TMZ), and later, an adjuvant treatment, again mainly with TMZ^6–8^, the well-known Stupp’s protocol^9^. Currently, more approaches are considering less aggressive treatments in elderly patients, who are treated by Perry’s modification (3 weeks chemoradiotherapy) or by chemotherapy alone^10–12^. It has been reported that median overall survival rates for the standard trimodal therapy is around 14 months^13^. In spite of this intensive therapy, disease relapse is very common, with a fatal outcome expected within 1 year^14^. That is believed to be the result of the significant tumor heterogeneity^15,16^, which complicates treatment, and also due to TMZ resistance mechanisms.

TMZ methylates several DNA nitrogenous bases. The most relevant damage is directed at *O*6-methyl-guanine (O6-meG) and at *N*7-methyl-guanine (N7-meG) or N3-methyl-adenine (N3-meA)^17^. The former is the most cytotoxic effect and can only be repaired by the enzyme O6-methylguanine DNA methyltransferase (MGMT), considered to be mainly responsible for chemo-resistance to TMZ^18–20^. However, several works show the important role of the base excision repair system (BER), which stems from the *N*-methylpurine-DNA-glycosylase (MPG) enzyme^21^, removing the most frequent damage (N7-meG and 3-meA). Furthermore, several studies determined MGMT and MPG gene expression in tumors and compared it with the corresponding normal tissue. In paired samples of brain, the results revealed that expression levels of both DNA repair enzymes were significantly higher in glioma tissues than that in non-neoplastic brain tissues^22,23^. This is in accordance with the available data registered in The Human Protein Atlas^24,25^. According to that, expression levels of MGMT and MPG in normal brain were found to be low and non region-specific. This demonstrates the multifactorial nature of DNA repair mechanisms^26,27^. Since resistance mechanisms are still poorly understood, there is an urgent need for improved treatment and alternative treatment regimes. Thus, we propose the study of the potential joint role of MGMT and MPG as targets in anti-resistance therapy to improve the therapeutic efficacy of alkylating agents. It is noteworthy that there is no scientific literature analyzing simultaneously these DNA repair enzymes in the context of TMZ resistance. With that in mind, the purpose of present work was to investigate the association between mRNA expression of MGMT and MPG and the effect of TMZ on patient-derived astrocytic cells in order to improve understanding of resistance mechanisms to chemotherapeutic agents in high grade astrocytic tumors.

## Results

### Clinical factors

A total of 18 patients were included in the study and follow-up after surgery was performed. As shown in Table 1, eleven patients were male (61%) and 7 females (31%) with a mean age of 62.61 years (r: 40–80). The temporal lobe is the most frequently involved tumor location (61%), eleven cases were in the right and seven in the left hemisphere (Table 1). These data are in agreement with previous findings focused on the incidence of gliomas by anatomic location. In particular, studies performed by Ellingson et al.^28^ reported that right temporal lobe, right basal ganglia, and right subventricular zone corresponded to brain regions with a high frequency of tumor occurrence. Mean time from symptom onset to diagnosis was 21 days (r = 1 h to 90 days). Cognitive change was the most frequent presentation (8 patients, 44%) followed by seizures (4, 22%) and cranial nerve palsy (5, 27%). Eight patients (44%) showed more than one symptom during the first consultation. Karnofsky Performance Status (KPS) scale ranged from 60 to 100 (mean 79) at the time of diagnosis (Table 2). Only one patient was diagnosed by biopsy alone (6%), while the remainder underwent open craniotomies and tumor resection; in six cases (33%) total resection was performed and in eleven cases subtotal resection (61%) (Table 1). After undergoing surgery either for biopsy or resection, 56% patients were entered into the Stupp protocol, consisting of concomitant chemo-radiotherapy with adjuvant TMZ; the rest received concomitant chemo-radiotherapy (6 cases), TMZ alone (GB18) and radiotherapy alone (GB2). 50% patients died during the study period.

**Table 1:** Clinical and molecular information and chemotherapy regimen of malignant glioma patients.

Patient code	Gender	Age	Glioma type	Location	Type of surgical resection	Ki67 (%)	ATRX	p53	Treatment
GB1	M	80	GB, IDH-wt	L; parietooccipital	Subtotal	15	Not mutated	Not mutated	RT/TMZ
GB2	M	52	GB, IDH-wt	L; frontoparietotemporal	Biopsy	25	Not mutated	Not mutated	RT
GB3	F	57	GB, IDH-wt	L; parietal	Total	40	Not mutated	Mutated	RT/TMZ
GB4	M	44	GB, IDH-wt	L; temporal	Total	10–12	Not mutated	Not mutated	RT/TMZ
GB5	M	52	AA, IDH-wt	L; temporal	Subtotal	67	Mutated	Not mutated	RT/TMZ
GB6	M	66	GB, IDH-wt	L; frontal	Subtotal	40	Mutated	Mutated	RT/TMZ + TMZ
GB7	M	65	GB, IDH-wt	R; frontal	Subtotal	5–10	Not mutated	Not mutated	RT/TMZ + TMZ
GB8	F	62	GB, IDH-wt	R; temporo-parieto-occipital	Total	40–50	Not mutated	Not mutated	RT/TMZ + TMZ
GB9	F	59	GB, IDH-wt	R; occipital	Subtotal	20	Not mutated	Mutated	RT/TMZ + TMZ
GB10	M	70	GB, IDH-wt	L; temporal	Total	70	Mutated	Mutated	RT/TMZ + TMZ
GB11	M	78	GB, IDH-wt	R; temporal	Subtotal	20	Mutated	Mutated	RT/TMZ + TMZ
GB12	F	77	AA, IDH-wt	R; frontal	Subtotal	5–10	Not mutated	Not mutated	RT/TMZ + TMZ
GB13	M	78	GB, IDH-wt	R; temporal	Subtotal	10	Mutated	Mutated	RT/TMZ + TMZ
GB14	F	51	AA, IDH-wt	R; temporal	Subtotal	30	Mutated	Not mutated	RT/TMZ + TMZ
GB15	M	40	GB, IDH-wt	R; temporal	Total	35–40	Not mutated	Mutated	RT/TMZ + TMZ
GB16	M	74	GB, IDH-wt	R; temporo-occipital	Total	10–12	Not mutated	Not mutated	RT/TMZ
GB17	F	70	GB, IDH-wt	R; frontal-parietal	Subtotal	5–8	Not mutated	Not mutated	RT/TMZ
GB18	F	52	GB, IDH-wt	R; frontal–temporal	Subtotal	70	Not mutated	Mutated	TMZ

Clinical and molecular data of individual patients concerning age (at time point of surgery), gender (M = male; F = female), glioma type (AA = anaplastic astrocytoma; GB = glioblastoma; IDH = isocitrate dehydrogenase; wt = wild type), tumor localization (L = left hemisphere; R = right hemisphere), type of surgical resection, results of immunohistochemistry; percentage of cell proliferation marker Ki67, mutation or loss of ATRX and p53 expression, and treatment (RT = radiotherapy; TMZ = temozolomide) of patients.

**Table 2:** Relevant symptoms, Karnofsky Performance Status scale and survival of high-grade glioma patients.

Patient code	Symptoms	Time to diagnosis (days)	KPS scale	Survival (months)
GB1	Cognitive disorder, seizures	60	90	† 9
GB2	Motor deficit, speech disorder	2	60	† 6
GB3	Motor deficit, speech disorder	7	60	† 18
GB4	Seizures	1	100	41
GB5	Cranial nerve palsy	15	80	† 31
GB6	Seizures	90	80	† 15
GB7	Behavior disturbance	30	70	† 13
GB8	Tumor bleeding	1	80	31
GB9	Cognitive disorder, cranial nerve palsy	21	80	25
GB10	Cognitive disorder, cranial nerve palsy	60	70	21
GB11	Sensory deficit	7	90	† 7
GB12	Cognitive disorder, seizures	30	80	23
GB13	Cognitive disorder	7	90	20
GB14	Cranial nerve palsy	15	90	20
GB15	Tumor bleeding	0.17	90	16
GB16	Cognitive disorder, cranial nerve palsy	21	60	† 5
GB17	Cognitive disorder	7	70	† 7
GB18	Cognitive disorder, impaired consciousness	7	60	13

Clinical data of individual patients concerning symptoms, time between initial symptoms to diagnosis, Karnofsky Performance Status (KPS) scale and survival in months († = patient died) of patients.

In all cases, histological examination confirmed a wild-type IDH1. These primary tumors, that account for 90% of all glioblastomas, are characterized by being more aggressive than secondary glioblastomas. In addition, immunohistochemical studies were performed by the neuropathology service in order to determine Ki67, ATRX and p53 protein expression. Cell proliferation marker ki67 is represented as the percentage of immunoreactivated cells. The lowest ki-67 value was 5–8% and the highest was 70%. From the analysis of tumor tissues, loss of expression (or mutated form) was observed in 6 (36,6%) and 8 (44%) cases for ATRX and p53, respectively (Table 1).

### mRNA expression levels of MGMT and MPG in patient-derived glioma cells

MGMT promoter methylation status seems to be a prognostic predictor in patients with glioblastoma treated with alkylating chemotherapy, such as TMZ^29^. However, recent work has shown that MGMT methylation does not always reflect gene expression^30^. Based on that, we aimed to analyze MGMT and MPG expression in the 18 low-passage primary astrocytic cells derived from patients by measuring the amount of mRNA associated with these DNA repair enzymes by means of real time reverse transcription polymerase chain reaction (real time RT-PCR) . On the other hand, it should be pointed out that, in order to preserve the intratumoral heterogeneity, as well as the molecular characteristics of the tumor, the primary cultures, used for expression and viability analysis, were established from the whole resected specimens.

**Figure 1:**
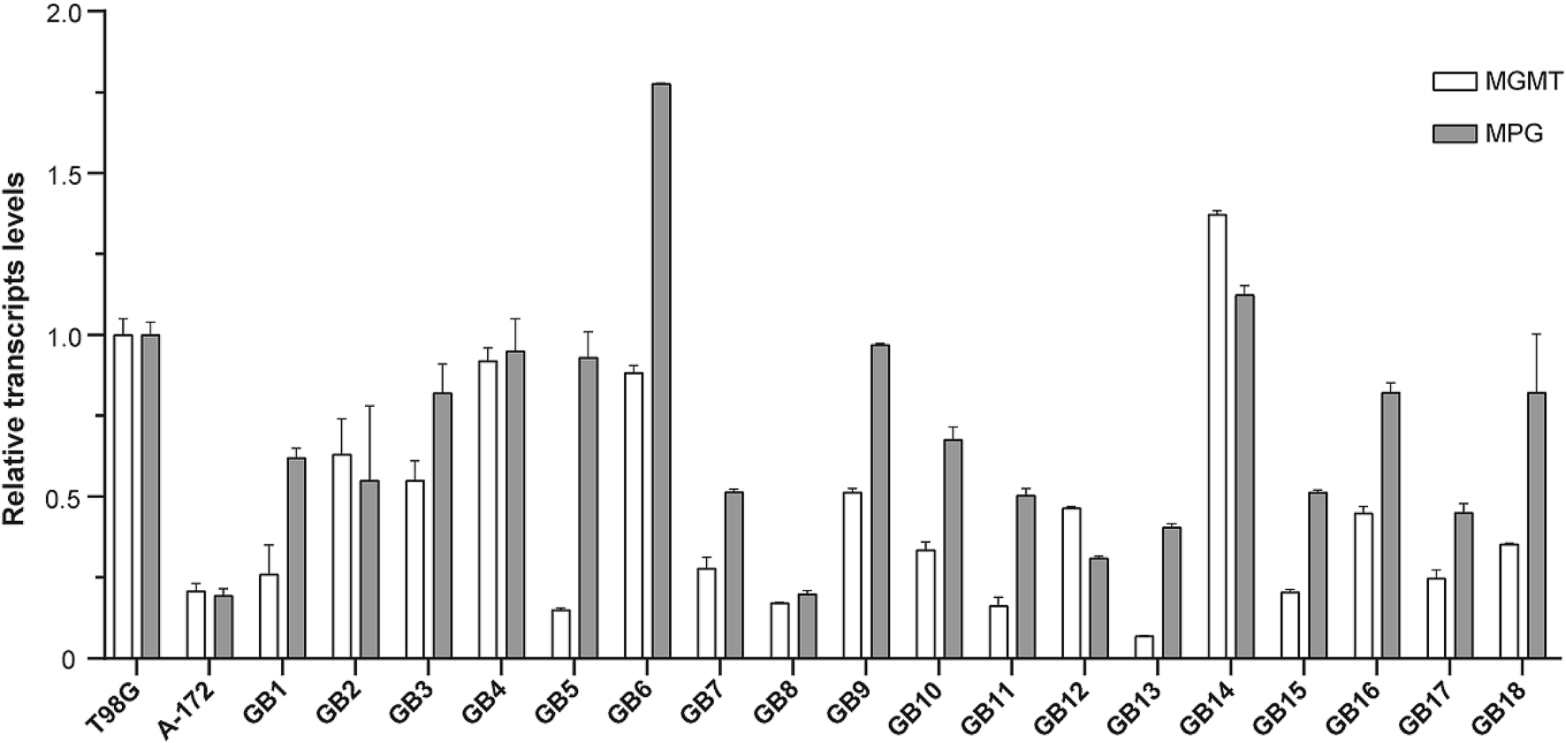
Relative mRNA expression levels of MGMT and MPG in human glioma cell lines (T98G and A-172) and patient-derived glioma cells. Primary cultures were established from the whole resected tumor after surgery. Once glioma cells reached confluence, total RNA was extracted and real-time RT-PCR analyses were performed to measure mRNA levels of the DNA repair enzymes. The histogram shows the MGMT and MPG transcripts amount relative to mRNA levels in T98G, a TMZ resistant-glioma cell line. A-172, a TMZ sensitive cell line, shows the lowest levels of MGMT and MPG. The MGMT mRNA was differentially expressed in primary glioma cells, whereas high mRNA levels of MPG were detected in most cell cultures. All of them had MPG values higher than A-172. Data are presented as mean ± SD; n = 2 (duplicates).

After performing the PCR experiments, the relative mRNA levels of MGMT and MPG were calculated by double delta Ct analysis^31^ using T98G, a well-known TMZ resistant established glioma cell line, as a reference with relative value 1, meaning this cell line exhibits high MGMT and MPG expression levels. In addition, A-172, a TMZ-sensitive established glioma cell line, was used as control of low gene expression of these DNA repair enzymes. The results revealed that the expression of MGMT and MPG in most patient-derived gliomas cells was lower than detected in control (T98G) cells. Most interestingly, we found that 90% of cell cultures showed high expression of MPG with values above 0.4–0.5, whereas mRNA levels of MGMT were significantly different for each glioma primary culture. Indeed, the latter exhibited high levels (above 0.5) in only 30% of cases: GB2, GB3, GB4, GB6 and GB14 (Fig. 1).

### TMZ sensitivity in primary glioma cell cultures

**Figure 2:**
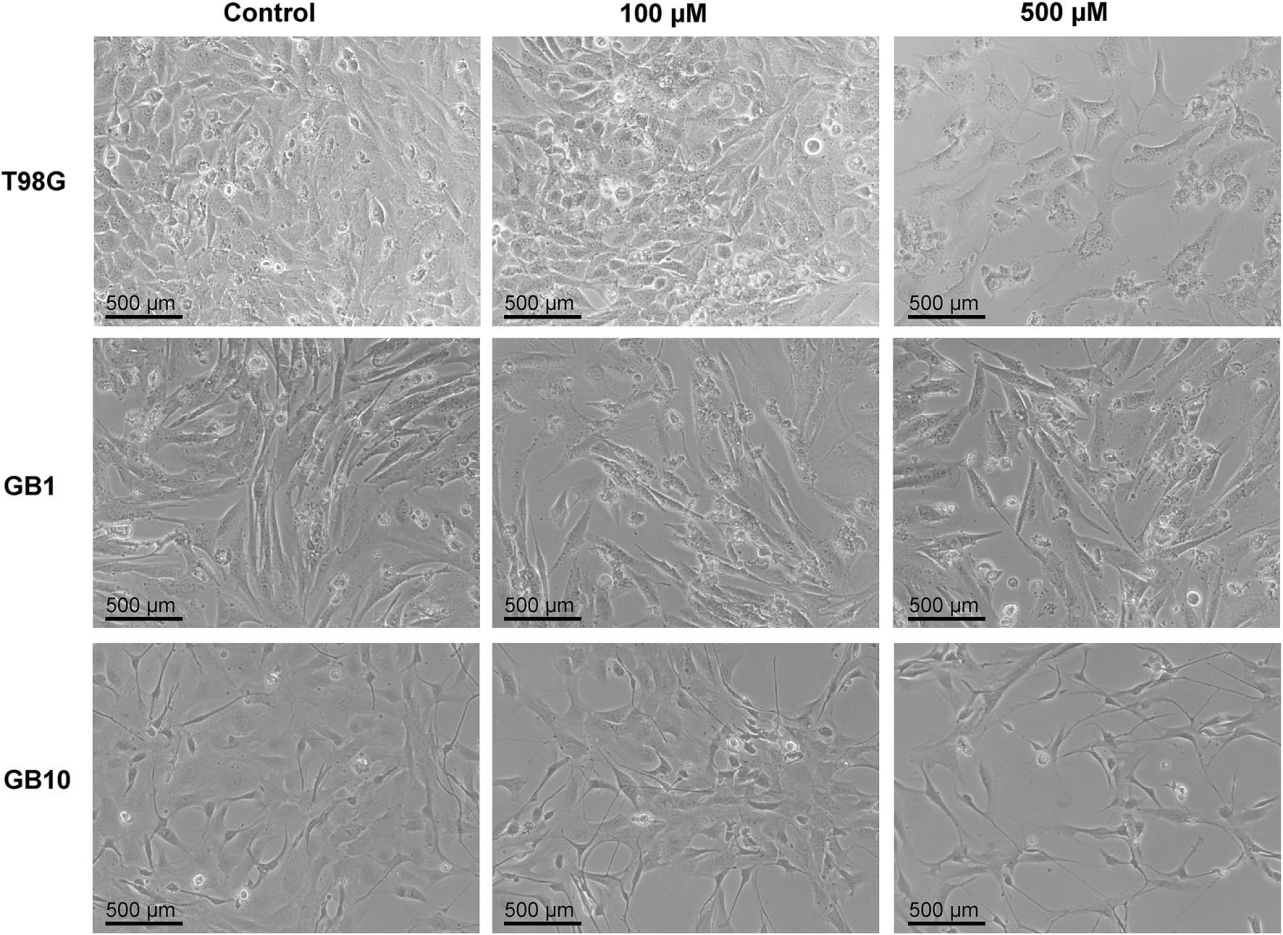
Representative photomicrographs of the morphological alterations displayed in TMZ-treated glioma cells. T98G cell line and primary glioma cells (GB1 and GB10) were cultured for 5 days with either dimethylsulphoxide vehicle (control) or 100 µM or 500 µM of TMZ. Glioma cells that were exposed to the highest dose of TMZ exhibited minimal morphological alterations indicative of a rounder shape and impaired cellular adhesion to the cell culture plate and a reduced cells number compared with TMZ-untreated cells. The images were captured using an inverted phase contrast microscope (X10 magnification, scale bar: 500 µm).

Next, the effect of TMZ on viability of patient-derived glioma cells was evaluated using optical microscopy and MTT assay. Microscopic examination showed that primary cultures exposed to TMZ were morphologically quite similar both to those treated with vehicle control (DMSO) and to T98G, a TMZ resistant cell line, treated with the same concentration of TMZ. At low doses of TMZ, treated cells remained large in number, attached to the bottom of the well, with fusiform shape and glossy appearance, which indicates a high proliferative capacity. However, a small proportion of round shaped cells, which were both smaller in size and number, was observed with 500 µM TMZ treatment, suggesting that exposure to the highest dose of TMZ for 5 days resulted in some impairment of cell viability and proliferation of primary cultures and T98G (Fig. 2).

**Figure 3:**
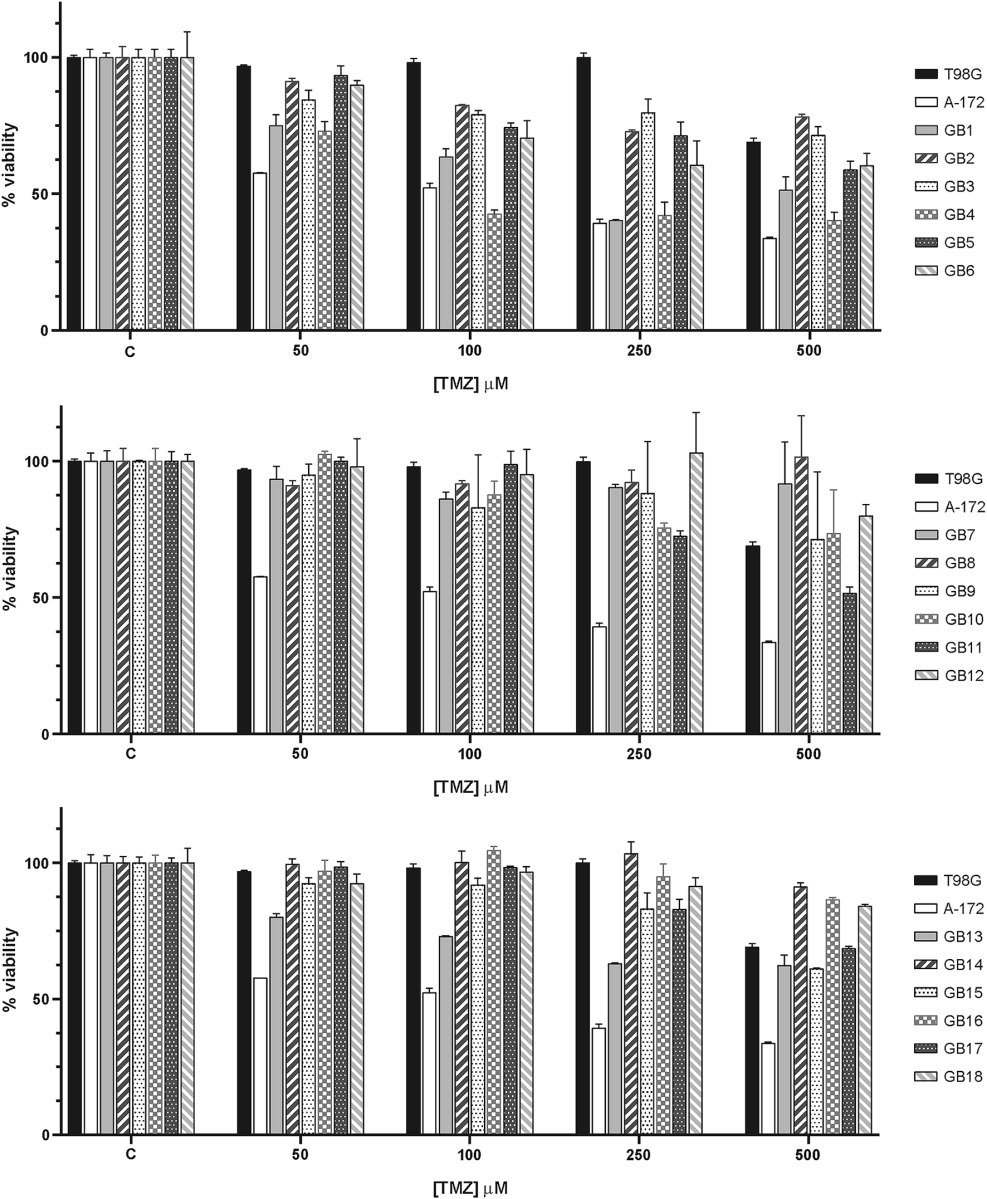
TMZ-induced antitumor effects on human glioma cell lines (T98G and A-172) and primary glioma cells. Cells were treated with either dimethylsulphoxide vehicle (C) or increasing concentrations of TMZ (50–500 μM) for 5 days and, then, cell viability was assessed by the MTT assay. Under exposure with the highest TMZ concentration, T98G showed a high resistance to TMZ, keeping 70% cells alive, while only 30% A-172 cells survived. Cell viability of all primary cultures remained over 40–50%. Data are presented as mean ± SD from three independent experiments.

In addition, MTT assays were performed to determine more accurately the viability of patient-derived glioma cells treated with the chemotherapeutic agent TMZ. Five concentrations were analyzed and subsequently its effect was tested. In all cases a similar response to the drug was seen. TMZ exerted slight toxicity on the patient-derived glioma cells, as compared with vehicle control-treated cells. As shown in Fig. 3, a minor and non-significant inhibition of cell viability was observed five days after TMZ therapy. In fact, more than 60% of the cells from almost all primary cultures remained alive at the highest concentrations of TMZ (500 µM), as observed with TMZ treatment of resistant control cell, T98G. As expected, the most significant reduction in viability was detected in TMZ-treated A-172, a well-know TMZ sensitive established glioma cell line (Fig. 3). The IC50 value of this drug in patient-derived glioma cells after 5-day treatment was estimated at 400–700 µM.

### Discussion

TMZ, an oral alkylating agent, is the main chemotherapeutic agent that shows clinical efficacy, in conjunction with surgery and radiotherapy, for glioblastoma^9^. Nevertheless, resistance to TMZ is a major obstacle to successful treatment of this cancer^32^.

Although elevated MGMT expression has been associated with resistance to TMZ, several studies suggest N methylpurine DNA glycosylase (MPG), an enzyme that repairs lesions produced on N7-meG and N3-meA by TMZ, plays an important role in that resistance^33,34^. In fact, many studies have shown that low levels of MGMT in glioblastomas were sufficient to confer TMZ resistance^27,35^, suggesting the existence of a MGMT-independent mechanism for TMZ resistance.

Our study aims at improving understanding of the role MPG plays in chemo-resistance. We found no correlation between MGMT mRNA levels and response to TMZ in the analysis of 18 patient-derived glioma cells. Interestingly, high levels of MPG expression displayed by nearly 90% of primary cultures were associated with TMZ resistance to a greater extent than MGMT expression. Regarding the resistance to TMZ of cases GB8 and GB12 (11% of the total), which both exhibit low MPG, and also MGMT, expression levels, we suggest that a wild-type pattern (absence of mutations) of important growth-control genes, ATRX and p53, observed by the immunohistochemical analysis, could explain why these cells remained alive at high doses of TMZ. Based on the review of DNA repair pathways in gliomas published by Yoshimoto et al.^36^, when MGMT and MPG are inactive, alkylated bases induced by TMZ are not repaired thereby causing a replication stall and collapse of the replication fork, which ultimately induce double-strand breaks (DSB). If DSBs remained unrepaired, this type of DNA damage triggers death in tumor cells. However, it could be repaired by ATM kinase, whose activation is modulated by ATRX wild type^37^, preventing cells from dying. On the other hand, the role of p53 has been viewed as a double-edged sword depending on the severity of damage. It is noteworthy that p53 relays a wide range of pro-survival signals like cell cycle arrest allowing the cells to repair the damage^38^. Thus, we propose that in the presence of low levels of MPG and MGMT, as is the case with GB8 and GB12 cells, the generated DSBs by TMZ treatment could be fixed by ATRX activated ATM during cell cycle arrest caused by p53, which would lead to chemoresistance.

Taken together, our molecular and cellular results suggest that MPG may be implicated in conferring drug resistance to glioblastomas harboring mutations in either ATRX or p53 or both, which account for around 60% of all glioblastomas. In agreement with the hypothesis, that MPG might be an important modulator of TMZ resistance in glioblastoma, it has been demonstrated that high levels of nuclear MPG expression detected in tumor samples of patients with glioblastoma correlated with poorer overall survival rates compared with patients who do not express the enzyme^27^. Similar results were reported in the study carried out by Liu et al.^23^ which examined the expression of the MPG gene in glioma samples with different WHO grades and its association with patient survival. Findings from that investigation showed that the expressed levels of the MPG gene increase as tumors progress from grade I to grade IV gliomas according to the results of real-time PCR, and that the survival rate of MPG-positive patients was significantly lower than that of MPG-negative patients. In agreement, clinical data registered in our study showed that nearly 40% of patients had died before 15 months (Table 2). We further suggest that the analysis of expression level of both MGMT and MPG in tumor samples after surgery or biopsy might be used to predict effectiveness of TMZ treatment in malignant glioma patients.

**Figure 4:**
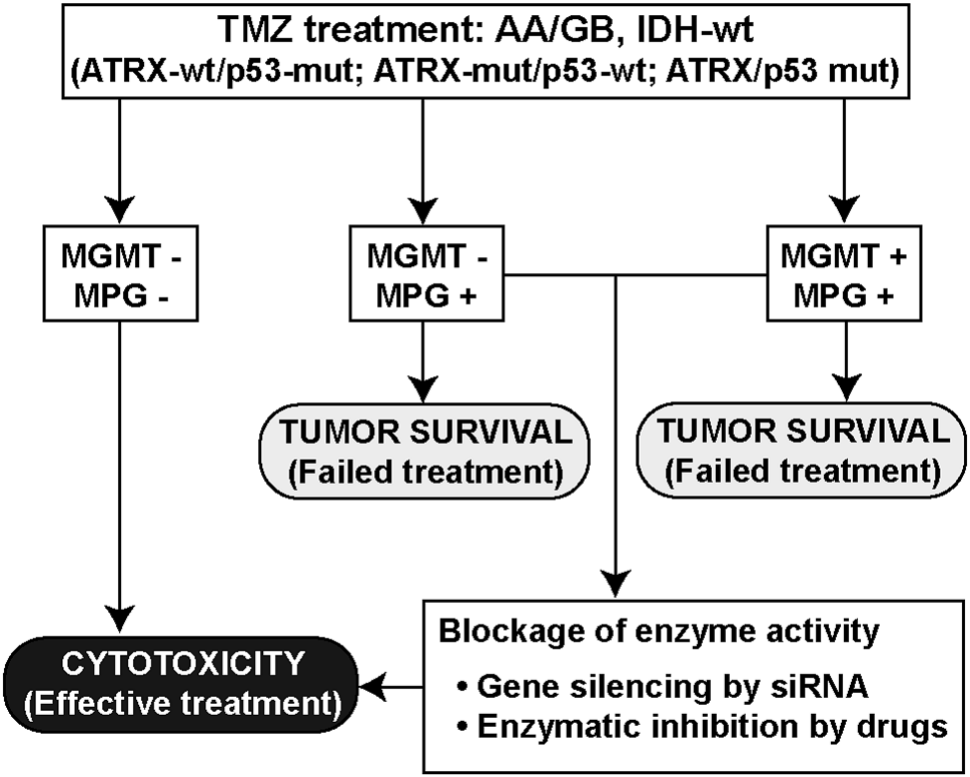
Proposed strategy for TMZ chemotherapy sensitization in malignant glioma. Direct inhibition of MGMT and MPG DNA repair enzymes is proposed as possible therapeutic approach to overcome TMZ resistance in high grade glioma. The enzymes inactivation could be carried out with siRNA gene silencing techniques or using active site- directed irreversible inhibitors.

In light of results obtained in this work, we propose the dual inhibition of MGMT, responsible for removing O6-methylguanine, and MPG, which initiates the BER pathway by excising N7, N3 methyl purines, as a strategy to overcome TMZ resistance in those group of glioblastomas with the following signature: ATRX-wt/p53-mut, ATRX-mut/p53-wt and ATRX-mut/p53-mut (Fig. 4), which represent, as mention before, approximately 60% of all glioblastomas. Such inactivation could be carried out by a gene silencing approach using nanoparticle platforms for siRNA delivery or by administration of drugs that block enzymatic activity. In this way, gliomas would be more susceptible to TMZ treatment, likely improving patient survival.

Due to the important role of the DNA damage response in the efficacy of chemotherapies utilizing alkylating agents for brain tumors, over the last decades, several inhibitors of DNA repair enzymes have been examined in preclinical and clinical studies. 06-BG, an O6-guanine derivative, is so far the most extensively studied direct MGMT inhibitor. This potent agent has been shown to reverse therapeutic resistance to TMZ by modulating MGMT expression in a variety of human tumor cell lines and xenograft models that include brain, melanoma, prostate and colon cancers^39^. However, several clinical trials with O6-BG and related compounds combined with the standard treatment have reported that this experimental therapy did not significantly influence the overall response rate and the median time to disease progression^40^. Accordingly, inhibition of the BER pathway has recently been proposed as an alternative strategy to enhance TMZ activity, due to the fact that blocking BER leads to the accumulation of repair intermediates that induce energy depletion-mediated cell death^41^. Studies conducted by Goellner et al.^41^ revealed that inhibition of both BER and NAD + biosynthesis significantly sensitizes glioma cells with elevated expression of MGMT, a genotype normally associated with TMZ resistance. Similarly, it has been demonstrated in glioma cells that MPG modulates BER inhibition-induced potentiation of TMZ in a Polβ-dependent manner^42^. In line with aforementioned studies, Bobola et al. reported that antisense suppression of MPG in human glioma cell lines induced sensitization to other alkylating agents such as methyllexitropsin^43^.

We acknowledge that the generalization of the results might be limited and a further study in a larger cohort of patients diagnosed with high grade glioma would be necessary. Additionally, preclinical and clinical studies are required to provide convincing evidence showing that inhibition of MPG alone or in combination with the MGMT inactivation enhances TMZ activity and produces a clinical benefit for patients with gliomas. Nevertheless, our findings, showing that MPG may be associated with TMZ resistance in human glioblastoma cells, could encourage and enable further research related to development of new therapeutic approaches to TMZ resistance using the enzyme MPG as a possible target along with MGMT.

## Methods

### Patient cohort and data collection

Samples were collected from patients diagnosed with glioma by the Neurology, Neurosurgery and Oncology departments of Complejo Hospitalario Universitario Albacete (Spain). Written informed consent was obtained from all patients according to the 1964 Declaration of Helsinki and its later amendments, and all the procedures were approved by the Human Ethics Committee of this Hospital (Committee´s reference: 03/2014). In the case of death, the signed informed consent was obtained by the next of kin or by a legal representative. The study protocol was performed according to relevant institutional and national guidelines and regulations.

Eighteen patients with anaplastic astrocytoma (AA), and glioblastoma (GB) all IDH-wild-type were selected using WHO criteria for tumor classification^2,3^. Histological examination of tumor samples was conducted by the neuropathology service; pathology reports were electronically retrieved from the archives of this service. In addition, relevant clinical information was obtained from the hospital information system including tumor location, extent of resection, ki67 and p53 indexes, data regarding antitumor therapy, symptomatology, KPS scale and survival of patients with malignant astrocytic tumors (Tables 1, 2).

### Immunohistochemical studies

Immunohistochemical analysis for IDH (clone H09, 1:20 dilution of mouse monoclonal antibody, Dianova), ATRX (clone Ax1, 1:300 dilution of mouse monoclonal antibody, Dianova) and p53 (DO7, mouse monoclonal antibody, Dako, Agilent Technologies) was performed on 5-μm-thick slides of tumor tissue using an automated immunostainer Dako Omnis following the manufacturer’s protocol.

IDH1 staining analysis was as follows: a strong cytoplasmic immunoreaction product was scored positive (mutant protein expression, variant R132H). ATRX staining analysis was as follows: nuclear ATRX loss (mutant protein expression) was scored negative if tumor cell nuclei were unstained. p53 staining was interpreted in two tiers: nuclear staining in more than 10% of the tumor cells was considered positive, samples without any nuclear staining of tumor cells (complete absence) were interpreted as negativity.

### Primary cultures and cell line

High grade glioma cells were obtained from patient specimens collected from the Neurosurgery department. The collected fresh tissue from resected gliomas was placed in sterilized tubes containing cold Earle’s Balanced Salt Solution (EBSS, Gibco). Primary cell cultures were established by mechanical division and enzymatic digestion with collagenase I and dispase II to a final concentration of 1 mg/ml in EBSS, for 45 min at 37 °C. After that, the pieces were completely disaggregated by gently pipetting and digested for 15 min with trypsin at 37 °C. Then, minced samples were centrifuged, cell pellets were suspended in growth medium RPMI 1640 (Gibco) supplemented with 20% heat inactivated fetal bovine serum (FBS, Gibco), 1% Penicilin/Streptomycin (P/S; Lonza) and 4 mM of glutamine (Lonza), and incubated at 37 °C and 5% CO_2_. Medium was replaced every 72 h until the cultures were confluent. Patient-derived astrocytic cells were continuously cultured until the experiments were carried out.

Human glioblastoma T98G and A-172 cell lines were obtained from American Type Culture Collection (ATCC CRL-1690 and CRL-1620, respectively, Rockville, MD, USA). T98G and A-172 cells were maintained in the same media conditions, RPMI 1640 medium supplemented with 20% FBS, 1% P/S, 4 mM of glutamine at 37 °C in a humidified atmosphere containing 5% CO_2_, as those for patient-derived glioma cells.

### Real time RT-PCR

Gene expression of MGMT and MPG was analyzed in patient-derived astrocytic cells and T98G and A-172 cell lines using real time RT-PCR. Total RNA was extracted from astrocytic cells using the RNeasy Mini Kit (50) (QIAGEN) and quantified by spectrophotometry with a NanoDrop ND-100. One microgram of total RNA was reverse-transcribed in a final volume of 20 μl with the RevertAid First Strand cDNA Synthesis Kit (Fermentas) using random primers, under the following reaction conditions: 25 °C for 5 min, 42 °C for 60 min, and 70 °C for 10 min. The cDNAs were then subjected to a real-time PCR analysis using Fast SYBR Green Master Mix in StepOnePlus Real-Time PCR system (Applied Biosystems) according to the manufacturer's instructions. 50 ng of each cDNA was used to perform the real-time PCR. The following settings were used for the real-time PCR reaction: an initial step of 10 min at 95 °C, followed by 45 cycles of 15 s at 95 °C and 1 min at 60 °C.

The sequences of the oligonucleotides used corresponded to MGMT and MPG target genes. Reverse and forward primers were used at a concentration of 0,3 µM and their sequences were as follows: MGMT1 forward: 5′-GTCGTTCACCAGACAGGTGTTA-3′ and reverse 5′-ACAGGATTGCCTCTCATTGCTC-3′; MGMT2 forward 5′-CCTGGCTGAATGCCTATTTC-3′ and reverse 5′-GATGAGGATGGGGACAGGATT-3′; MPG1 forward 5′-GTCCTAGTCCGGCGACTTCC-3′ and reverse 5′-CTTGTCTGGGCAGGCCCTTTGC-3′; MPG2 forward 5′-CTGTGCCAGGCCCTGGCCATCA-3′ and reverse 5′-CACTCTGTCGACCACACTGACC-3′. As internal controls for gene expression normalization, mRNA levels of β-actin and β-tubulin were measured using the following primers: β-actin forward 5′-AAGATCATTGCTCCTCCTG-3′ and reverse 5′-CGTCATACTCCTGCTTGCTG-3′; β-tubulin forward 5′-CTTCGGCCAGATCTTCAGAC-3′ and reverse 5′-AGAGAGTGG GTCAGCTGGGAA-3′.

The relative mRNA levels were calculated by the 2^−ΔΔCq^ method, using the cycle threshold (Ct) values of transcripts determined by StepOne Software v.2.1. The T98G cell line was used as reference, meaning it exhibits the maximum MGMT/MPG mRNA expression levels. β-actin was selected as a control for normalization. Amplicon purity and size were verified by melt curve analysis (melting program settings: 15 s at 95 °C, 1 min at 60 °C and 15 s at 95 °C). The samples were used in duplicate and the experiment was repeated twice.

### Cell viability assay

Human cell lines (T98G and A-172) and patient-derived glioma cells were seeded at a density of 2 × 10^4^ cells per well in a 12-well plate and were cultured in growth medium RPMI 1640 supplemented with 20% heat FBS, 1% Penicilin/Streptomycin and 4 mM of glutamine at 37˚C and 5% CO_2_. Twenty-four hours later, the cells were treated with increasing TMZ concentrations ranging from 50 to 500 µM. The controls consisted of equal dimethyl sulfoxide (DMSO) concentrations as needed for the dilution of TMZ therapy. Five days later, cell viability was assessed by MTT assay (Thiazolyl Blue Tetrazolium Bromide, Sigma). The MTT agent dissolved in DMEM (Dulbecco's Modified Eagle Medium without phenol red, Lonza), at a concentration of 5 mg/ml, was added to each well, followed by culture for 30 min–1 h at 37 °C. A blank solution was included in a well without cells, using only MTT in DMEM. The purple crystals were dissolved in 1 ml DMSO and the absorbance of the obtained solution was measured at a wavelength of 562 nm (555–690 nm) in a multi-well plate reader (SPECTROstar Omega spectrophotometer, BMG Labtech). All experiments were carried out in triplicate. Results were plotted as the mean values of triplicates from a representative experiment that was repeated independently at least twice. The dose of TMZ which inhibited glioma-derived cell proliferation by 50% (IC50) was estimated by plotting various concentrations of TMZ versus percentage of cell viability.

## Data Availability

The data that support the findings of this study are available on request from the corresponding author.
